# Long term COST-minimization analysis of robot-assisted hysterectomy versus conventional laparoscopic hysterectomy

**DOI:** 10.1186/s13561-019-0236-8

**Published:** 2019-06-18

**Authors:** María A. Martínez-Maestre, Lidia M. Melero-Cortés, Pluvio J. Coronado, Carmen González-Cejudo, Nuria García-Agua, Antonio J. García-Ruíz, Francisco Jódar-Sánchez

**Affiliations:** 1 Gynecology Unit, Virgen del Rocio University Hospital, Seville, Spain; 2 Women’s Health Institute, San Carlos Clinic Hospital, IdISSC, Madrid, Spain; 3 Health Economics & Rational Use of Drugs, Faculty of Medicine, University of Málaga, Málaga, Spain; 4 Pharmacoeconomics: Clinical and Economic Evaluation of Pharmaceutical Drugs and Palliative Care, Institute of Biomedical Research in Malaga (IBIMA), Málaga, Spain; 5 Biomedical Informatics, Biomedical Engineering and Health Economy, Institute of Biomedicine of Seville, IBiS/Virgen del Rocío University Hospital / CSIC / University of Seville, Seville, Spain

**Keywords:** Economic evaluation, Hysterectomy, Laparoscopy, Robotic surgery

## Abstract

**Background:**

The aim of this study is to carry out the economic evaluation, in term of a cost-minimization analysis that considers healthcare costs and indirect costs, of robot-assisted hysterectomy (RAH) compared with conventional laparoscopic hysterectomy (CLH) in female adults scheduled for total laparoscopic hysterectomy for benign conditions.

**Methods:**

Cost-minimization analysis based on an analytic observational study of prospective cohorts with a five-year time horizon. Eligible participants were all female adults scheduled for total laparoscopic hysterectomy for benign conditions at tertiary hospital. The economic evaluation was conducted from a Spanish National Health Service and societal perspective, including healthcare costs and indirect costs. The costs are expressed in Euros from the year 2015.

**Results:**

One hundred sixty nine patients were analyzed, 68 in the RAH group and 101 in the CLH group. Average cost for the RAH group was €8982.42 compared to €8015.14 for the CLH group (incremental cost €967.27; *p* = 0.054). Healthcare cost is the most important component of total cost and represents 86.4% for the RAH group and 82.3% for the CLH group. The difference of €1169 (*p* = 0.01) in the average healthcare cost is mainly due to the cost of purchasing and maintaining the equipment (difference of €1206.39 in favor of RAH; *p* < 0.005). With regard to indirect costs, for patients in the RAH group the costs associated with loss of productivity were lower (difference of €203.42; *p* = 0.17), while the cost of trips to the hospital was higher (difference of €1.98; *p* = 0.66) in respect to CLH.

**Conclusions:**

Our findings reveal similar effectiveness between RAH and CLH, although CLH is the more efficient option from the point of view of an economic analysis based on cost-minimization.

## Background

Hysterectomy is one of the most common major surgical procedures performed all over the world. Although hysterectomies were traditionally approached by either vaginal or abdominal route, technological advances have led to an increase in laparoscopic hysterectomy, because it has shown clear advantages and fewer complications [1]. Nevertheless, laparoscopic hysterectomy continues to lag well behind laparotomy, because the laparoscopic approach is affected by the surgeon’s skill level and the technical limitations of conventional laparoscopic instruments [1].

Robot-assisted surgery was developed to overcome some of the limitations of laparoscopic surgery. The stable, three-dimensional view, more range of motion and improved coordination facilitate to learn the operating technique, which has made this advanced laparoscopic surgical procedure accessible to surgeons who do not have advanced video-endoscopic training [1]. These are the main reasons why, since its introduction, robotic surgery has generated enormous public excitement and achieved an impressive market penetration.

Current evidence demonstrates neither statistically significant nor clinically meaningful differences in surgical outcomes between robotic and laparoscopic hysterectomy for benign disease [2]. On the other hand, cost analyses done in the last few years found that outcomes achieved using robotic techniques were not significantly better than those achieved using conventional laparoscopy and the variable cost was considerably higher with the first option [3, 4]. However, the available evidence is limited to perioperative outcomes.

The aim of this study is to carry out the economic evaluation, in term of a cost-minimization analysis that considers healthcare costs and indirect costs, of robot-assisted hysterectomy (RAH) compared with conventional laparoscopic hysterectomy (CLH) in female adults scheduled for total laparoscopic hysterectomy for benign conditions. To the best of our knowledge, this is the first study considering long-term results comparing both techniques from a societal perspective.

## Methods

### Study design

Cost-minimization analysis based on an analytic observational study of prospective cohorts with a five-year time horizon was applied.

### Study population

Eligible participants were all female adults scheduled for total laparoscopic hysterectomy for benign conditions between November 2007 and September 2011 at the “Virgen del Rocio University Hospital “. Inclusion criteria were as follows: elective surgery and uterine length ≤ 16 cm at vaginal ultrasound. Patients with malignancy, genital prolapse indicating vaginal hysterectomy, suspicion of adnexal malignancy, adnexal mass > 7 cm in maximum diameter, known or suspected endometriosis, extensive adhesions contraindicating a laparoscopic approach, and comorbidity contraindicating surgery were excluded. All patients signed a consent form according to local ethical requirements.

All patients were included consecutively and were assigned to either conventional video-assisted laparoscopy or da Vinci robotic surgery depending on the patient’s position on a hospital waiting list and the availability of the interdivisional robot on the scheduled surgery date. Neither the researchers nor the surgeons were able to intervene in the assignment. The follow-up were performing in Gynecology service.

### Surgical technique: minimally invasive surgery

Robotic-assisted technology allows surgeons to perform complex procedures more easily in compassion with conventional laparoscopy, because of 3D visualization, wristed instruments and intuitive movements.

The surgical technique was standardized and previously published [5]. Clermont-Ferrand uterine manipulator (Karl Storz Endoscopy, Tuttingen, Germany) was used. Pneumoperitoneum was established with a Verres needle. In both procedures, bipolar coagulation was used, but not vessel sealer. Colpotomy was performed by monopolar scissors and sutured with intracorporeal knotting.

### Cost-minimization analysis

Methods of economic evaluation have been developed to facilitate efficient resource allocation. Economic evaluation, synonymous of efficiency evaluation, is the comparative analysis of alternative courses of action in terms of both their costs and consequences. When the consequence or benefit of a health technology produces in routine clinical practice, it is measured through effectiveness. If two or more procedures are clinically equivalent, the only aspect that should be considered and compared are the costs, without taking into account the health outcomes [6].

To demonstrate the equivalence of the two procedures, this study analyzed the health outcomes in terms of survival and major complications that required surgical intervention in the five years following the original procedure. The economic evaluation was conducted from a societal perspective, including healthcare costs and indirect costs. Costs are expressed in Euros from the year 2015.

### Healthcare resources and healthcare cost

Healthcare resources included in the analysis were Accident and Emergency (A&E) department consultations for benign gynecological pathologies, specialist consultations for benign gynecological pathologies, hospitalizations associated with the primary intervention or for subsequent surgical procedures associated with the original one, material and equipment. Information on the use of healthcare resources was extracted from hospital information system databases. Healthcare costs were calculated based on the use of healthcare services:

- A&E department consultations and specialist consultations costs were estimated using the price list published by the Andalusian Public Health System [7].
- Hospitalizations cost was estimated based on the diagnosis related groups (DRG) associated with the hospitalization (DRG 358: Uterine & Adnexa procedures for non-malignancy W CC; DRG 359: Uterine & Adnexa procedures for non-malignancy W/O CC) [8]. DGR is a unit of classifying patients by diagnosis, average length of hospital stay and therapy received.
- Material (disposable and reusable). For the CLH group this included disposable materials such as monopolar scissors (EndoShear), camera cover and ten times reusable instruments, such as forceps (both bipolar hemostasis forceps and fenestrated forceps) and trocars. In the RAH group, it included disposable materials such as covers (camera cover and two robotic arm covers); and reusable instruments such as Endowrist® forceps, monopolar scissors (Monopolar Curved Scissors); bipolar forceps (Maryland Bipolar Forceps) and trocars (Large Needle Driver). The costs of the material were calculated using market prices and for the reusable material, the number of uses was considered.
- Acquisition and maintenance of equipment. The equipment included the laparoscope video tower for the CLH group and the da Vinci robot for the RAL group. A useful life of 10 years was used to calculate the cost. The analysis did not include materials and instruments that are common to both techniques such as uterine manipulators (Clermont-Ferrand, Stortz®), Veress needles, reusable trocars (10 and 5 mm) and the irrigation-aspiration system.

### Indirect costs

Indirect costs were based on loss of productivity and transport to the hospital. The first one considers the time during which the patient is out of work on medical leave, from the date of the procedure until the patient resumes their normal activities. Analysis only considered loss of productivity for women, no caregivers. The calculation was based on the cost of labor from the National Statistics Institute [9].

It was assumed that patients travelled to the hospital in their own private vehicles. Estimated transport costs are based on the distance from the patient’s home to the hospital. The cost assigned was the amount of the compensation payable for the use of private vehicles [10].

### Statistical analysis

Descriptive analyses of the socio-demographic variables included calculating the mean, standard deviation, median and interquartile range for the quantitative variables, and absolute and relative frequencies for the qualitative variables. Bivariate analyses were performed to evaluate the differences between groups in terms of healthcare resources and costs. Quantitative variables were analyzed with the t-Student test or Mann-Whitney U test depending on the variable distribution; for qualitative variables were analyzed with the chi-squared test. Cumulative incidences, relative risk (RR) with 95% confidence interval (CI) were analyzed to compare difference in long-term patient outcomes between two groups. Statistical significance was set at *p* < 0.05.

To analyze the uncertainty of the base case results, a tornado diagram of the univariate sensitivity analysis was drawn, incorporating variations of 0% and 6% in the cost components.

## Results

A total of 169 patients were analyzed, 68 in the RAH group and 101 in the CLH group. The characteristics of the patients are shown on Table 1. No differences were found in relation to age, BMI, Charlson comorbidity index, prior abdominal surgeries or uterine weigh.

**Table 1:** Patient characteristics. Data are given as mean ± standard deviation or frequencies (%)

	CLH (*n* = 101)	RAH (*n* = 68)	*P* value
Age (years)	50.21 ± 9.36	48.03 ± 9.57	0.14
< 50 years	62 (61.39)	50 (73.53)	
≥ 50 years	39 (38.61)	18 (26.47)	
Comorbidity index	0.40 ± 0.76	0.26 ± 0.66	0.25
0	73 (72.28)	56 (82.35)	
1	20 (19.80)	8 (11.77)	
≥ 2	8 (7.92)	4 (5.88)	
Body Mass Index (kg/m^2^)	27.31 ± 4.48	28.16 ± 4.73	0.24
< 30 kg/m^2^	71 (70.30)	42 (61.76)	
≥ 30 kg/m^2^	30 (29.70)	26 (38.24)	
Without prior abdominal surgery	67 (66.34)	44 (64.71)	0.83
Prior abdominal surgery, mean	0.44 ± 0.70	0.44 ± 0.66	0.96
Caesarean^a^	6 (5.94)	5 (7.35)	
Appendectomy^a^	17 (16.83)	9 (13.24)	
Cholecystectomy^a^	8 (7.92)	3 (4.41)	
Tubal sterilization^a^	7 (6.93)	5 (7.35)	
Other^a^	6 (5.94)	8 (11.77)	
Weight of the surgical specimen (g)	208.54 ± 136.63	192.49 ± 105.80	0.43

*RAH* robot-assisted hysterectomy, *CLH*: conventional laparoscopic hysterectomy

^a^Multiple responses allowed in information of prior abdominal surgery

No patients died of both groups. Cumulative incidences of major complications related to the benign gynecological pathology requiring surgical intervention were 8.8% vs. 8.9% for the RAH and CLH groups respectively (*RR = 0,99; 95% CI: 0.37 to 2.65*). Taking into account that RR is close to 1, it suggests no difference in major complications risk (incidence in each group is the same with a difference of 0.1% over the 5-year time horizon; *p* = 0.984).

Therefore, according with evidence of systematic review [11], in this study it was assumed that the relevant health outcomes (survival and major complications) of both techniques are equal, what justifies the performance of a cost-minimization analysis.

### Healthcare resources and healthcare costs

For the 5-year period following the surgical procedure, hospital stays were reduced by an average of 0.90 days (2.76 ± 3.82 in the CLH group vs 1.87 ± 1.54 in the RAH; *p* = 0.07). The average number of consultation to the A&E department was 0.1 higher for RAH (0.63 ± 1.06 vs 0.53 ± 0.98 respectively; *p* = 0.52), while the average number of visits to specialists was 0.52 higher among patients in the RAH group (2.13 ± 2.20 vs 2.65 ± 2.32; *p* = 0.14). For complications associated with the benign gynecological pathology, no differences were observed between the two groups in the rate of patients requiring additional surgeries (8.9% in the CLH group vs. 8.8% in the RAH group; *p* = 0.98).

**Fig. 1:**
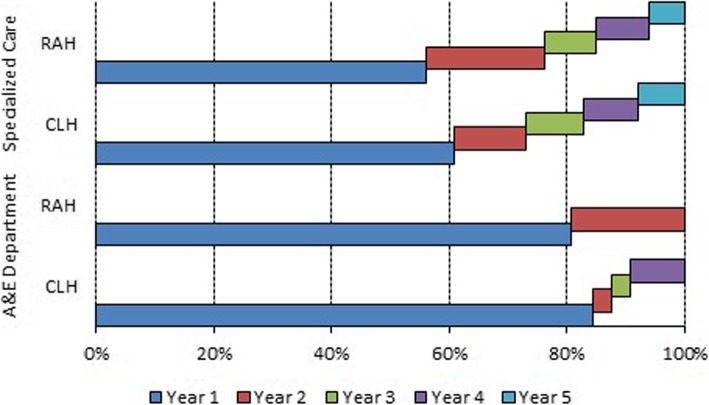
Distribution of A&E department and specialist visits

Figure 1 shows the consultations to both the A&E department and the specialists during the five years following the total hysterectomy. For both groups, the number of consultations to the A&E department was highest in the first year (80.6% in the RAH group vs. 84.4% in the CLH group). The patients in the RAH group accounted for the rest of the consultations to the A&E department visits during the second year (19.4%), although those visits by patients in the CLH group were more prolonged over the time: 3.1% in the second year, 3.1% in the third year and 9.4% in the fourth year.

The breakdown for visits to specialists was similar in both groups, with the main differences being observed in the first two years. The percentages of visits to specialists during the five years after surgery were 56.1%, 20%, 8.9%, 8.9% and 6.1% for the RAH group and 60.9%, 12.1%, 9.8%, 9.3% and 7.9% for the CLH group.

In the Table 2 are shown indirect and total healthcare costs. The healthcare cost for the RAH group was higher than for the CLH (*p* = 0.01). The breakdown of healthcare costs over the five-year period showed that they are concentrated almost entirely in the first year, accounting for 95.3% for the RAH group and 98.3% for the CLH group. The difference in the average healthcare cost in the first year was €912.86 (*p* = 0.02), being higher for patients in the RAH group. Analyzing the evolution of average healthcare costs over the rest of the years, we observed that in the second and third years the costs rose less for the patients in the CLH group by €243.36 (*p* = 0.04) and €72.66 (*p* = 0.23), respectively. In the fourth and fifth years they were €30.98 (*p* = 0.32) and €29.19 (*p* = 0.43) lower for the patients in the RAH group.

**Table 2:** Indirect and total healthcare cost (€). Data presented as Mean ± standard deviation; (Median; Q1 – Q3)

	CLH (*n* = 101)	RAH (*n* = 68)	*P* value
Healthcare cost	6593.33 ± 2766.09 (5574.93; 5450.66 – 6320.96)	7762.04 ± 2378.25 (6948.67; 6705.29 – 7972.32)	0.01
Hospital admissions	5235.10 ± 1269.43 (4877.15; 4633.77 – 5500.88)	5013.08 ± 1352.95 (4633.77; 4526.77 – 4877.15)	0.28
A&E Department	91.40 ± 153.63 (0; 0–144.24)	76.36 ± 142.01 (0; 0–108,18)	0.52
Specialized care	175.73 ± 119.99 (114,12; 114.12–168.70)	204.02 ± 126.79 (168.70; 114.12–223.28)	0.14
Hospital re-admissions	504.40 ± 2037.11 (0; 0–0)	456.72 ± 1614.06 (0; 0–0)	0.87
Material	82.66 ± 29.63 (74.54; 74.54–74.54)	301.43 ± 26.20 (293.76; 293.76–293.76)	< 0.001
Equipment	504,04 ± 180,69 (45.83; 45.83–45.83)	1710,43 ± 159,78 (900.00; 900.00–900.00)	< 0.001
Indirect cost	1421.82 ± 1194.90 (877.28; 617.35–2200.80)	1220.38 ± 727.67 (1308.02; 627.23–1330.74)	0.22
Productivity loss	1407.72 ± 1193.62 (871.20; 609.84–2178.00)	1204.31 ± 718.87 (1306.80; 609.84–1306.80)	0.21
Trips to the *hospital*	14.10 ± 23.25 (6.50; 3.02–12.74)	16.07 ± 34.16 (8.46; 3.73–17.48)	0.66
Total cost	8015.14 ± 3512.50 (6834.27; 6171.61 – 8292.46)	8982.42 ± 2611.54 (8246.69; 7821.47 – 9376.90)	0.054

*A&E* Accident and Emergency

### Indirect cost

The average loss of productivity for the five-year period was 13.82 ± 8.25 days for the RAH group and 16.16 ± 13.70 for the CLH group (difference of 2.36 days; *p* = 0.17). Of this total, it was obtained the same relationship when analyzing the loss of productivity after the initial surgery: 12.90 ± 7.31 days for the RAH group and 14.51 ± 11.12 for the CLH group (difference of 1.62 days *p* = 0.26).

The same differences were observed when analyzing the delay in returning to work by subgroups. Among patients requiring no additional surgery, the average was 12.95 ± 7.17 days for the RAH group and 14.21 ± 11.14 days for the CLH group (*p* = 0.43). Among patients that required additional surgery, the average was 22.83 ± 13.35 days for the RAH group (12.33 ± 9.35 days after the initial surgery and 10.50 ± 4.97 days after the additional surgery) and 36.11 ± 21.03 days (17.67 ± 11.00 days after the initial surgery and 18.44 ± 10.31 days for the CLH group after the additional surgery) (*p* = 0.20).

The average number of trips to the hospital during the 5 years following the original procedure was 8.56 ± 5.80 for the RAH group and 7.74 ± 5.67 for the CLH group (*p* = 0.36). Both groups had a similar average number of trips, except in the second year: 5.88 ± 2.47 for the RAH group vs. 5.88 ± 2.73 for the CLH group in the first year (*p* = 0.99); and 1.41 ± 2.37 for the RAH group versus 0.55 ± 1.27 for the HLC group in the second year (*p* = 0.003). There were no statistical differences in relation to indirect cost between RAH and CLH (Table 2).

### Cost-minimization analysis

From a societal perspective, including healthcare and indirect costs in the analysis, the average cost for the RAH group was €8982.42 compared to €8015.14 for the CLH group (incremental cost of €967.27; *p* = 0.054). In terms of the median, the total cost was €8246.69 for the RAH group and €6834.27 for the CLH group (a difference of €1412.42). Table 2 shows the healthcare and indirect costs included in the analysis.

Healthcare cost is the most important component of total cost and represents 86.4% for the RAH group and 82.3% for the CLH group. The difference of €1169 in the average healthcare cost is mainly due to the cost of purchasing and maintaining the equipment (a difference of €1206.39; *p* < 0.001). In addition, the costs associated with disposable and reusable material (an average difference of €218.78; *p* < 0.001) and specialist consultations (a difference of €28.29; *p* = 0.14) were higher in the RAH group. By contrast, for patients in the CLH group the costs associated with hospitalizations (a difference of €222.02; *p* = 0.28), consultations to the A&E department (a difference of €15.04; *p* = 0.521) and hospital re-admissions for additional surgeries (difference of €47.68; *p* = 0.87) were higher.

**Fig. 2:**
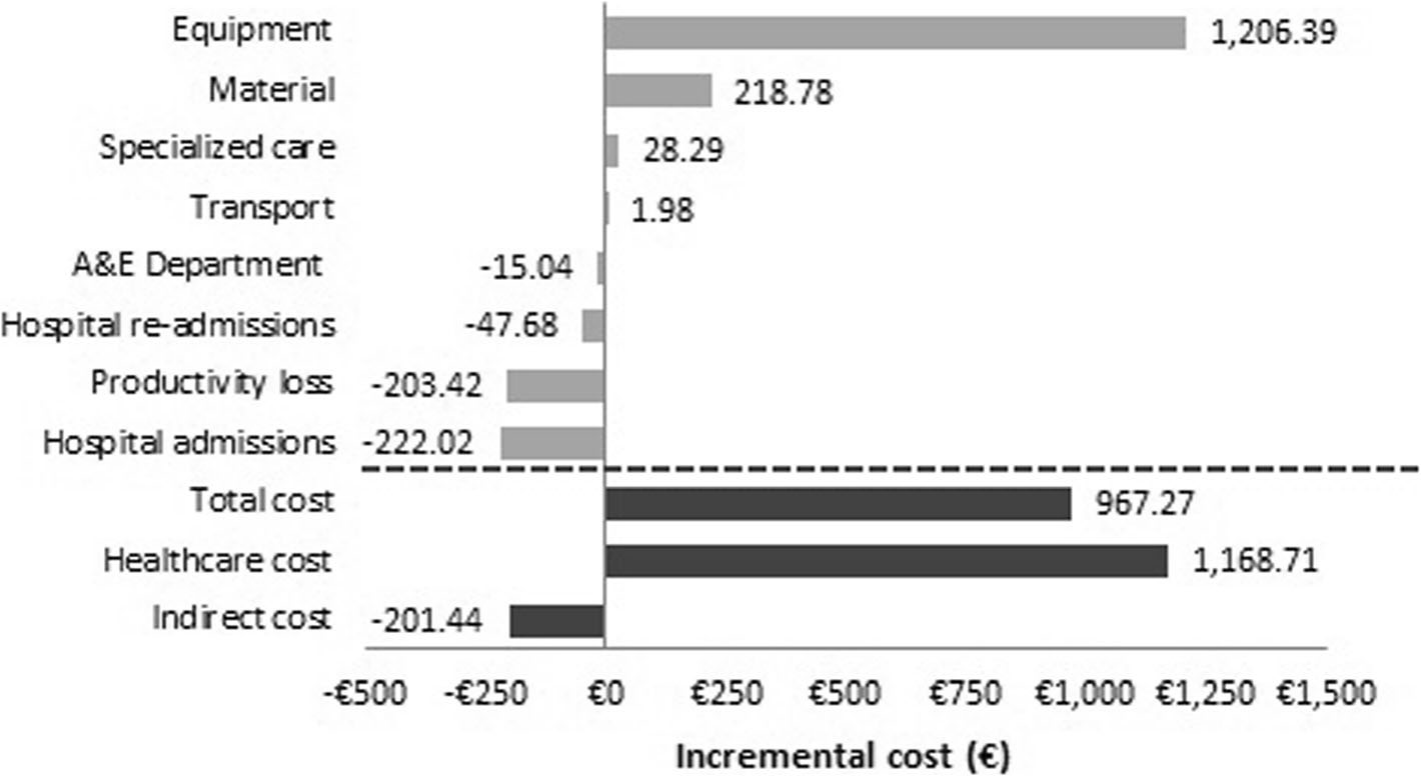
Incremental cost of RAH compared with CLH

**Fig. 3:**
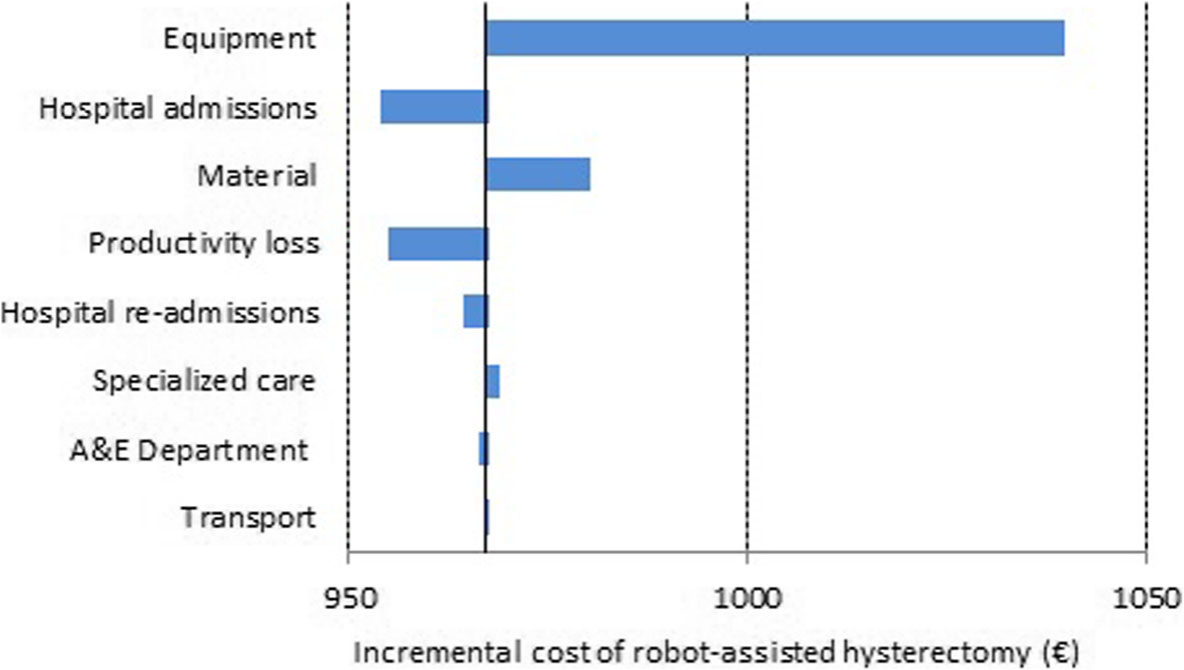
Sensitivity analysis

With regard to indirect costs, for patients in the RAH group the costs associated with loss of productivity were lower (a difference of €203.42; *p* = 0.17) while the cost of trips to the hospital was higher (a difference of €1.98; *p* = 0.66). Figure 2 shows the average incremental cost for RAH vs. CLH patients. Figure 3 show the results of the sensitivity analysis. Based on our findings, equipment demonstrated the largest impact on cost difference taking into account that this cost can significantly alter the results of the analysis, increasing the mean cost difference between RAH and CLH groups until €1040. According with base case results, variations in the equipment and material costs maximize the cost difference between groups and variations in the hospital admissions and productivity loss costs minimize the cost difference between groups.

**Fig. 4:**
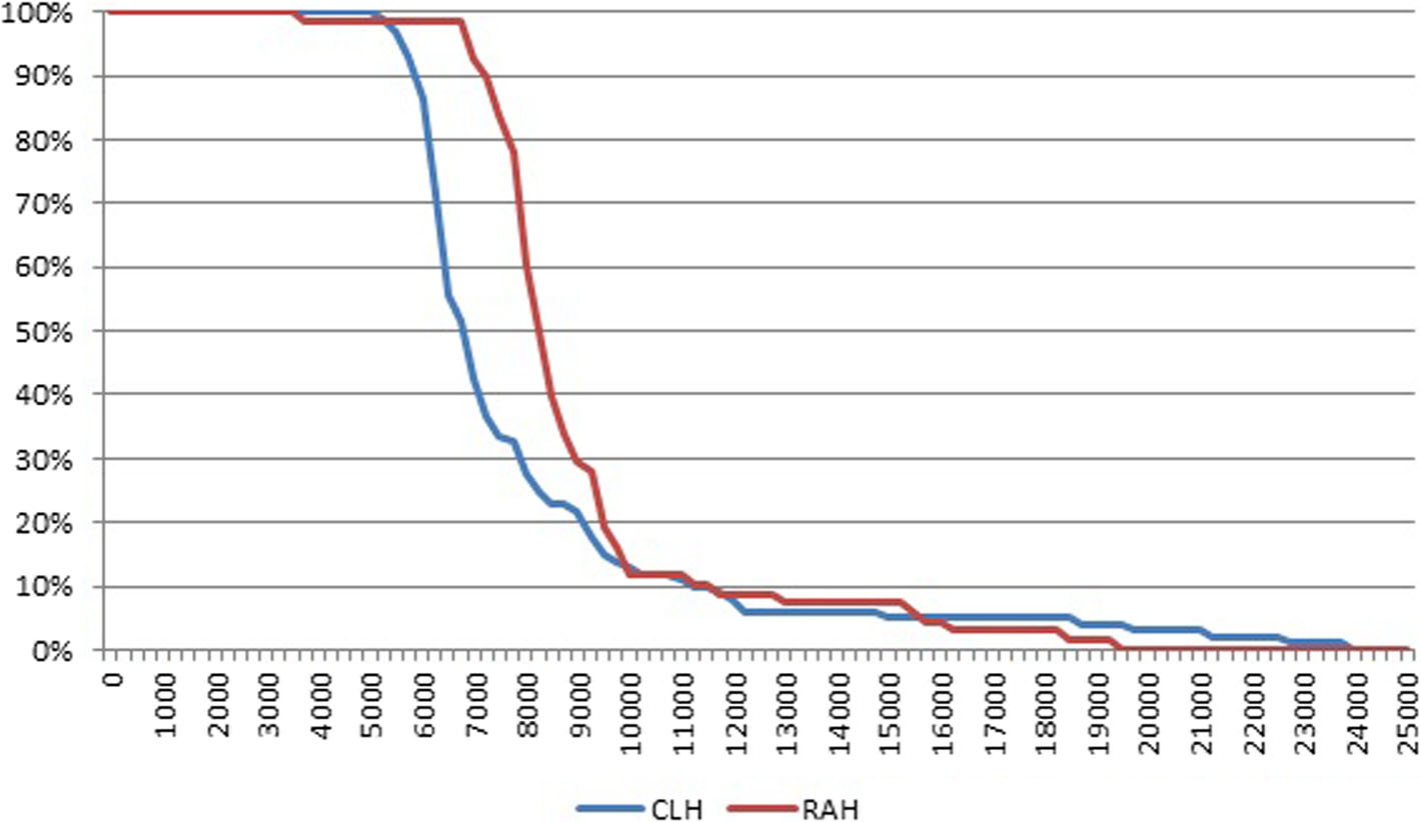
Breakdown of total average cost

Figure 4 shows a breakdown of the total average costs. There were differences between the two groups over the five-year study period (*p* = 0.01). An analysis of the breakdown by year showed that 98.2% and 95.3% of the total cost was concentrated in the first year for the CLH and RAH groups, respectively.

**Fig. 5:**
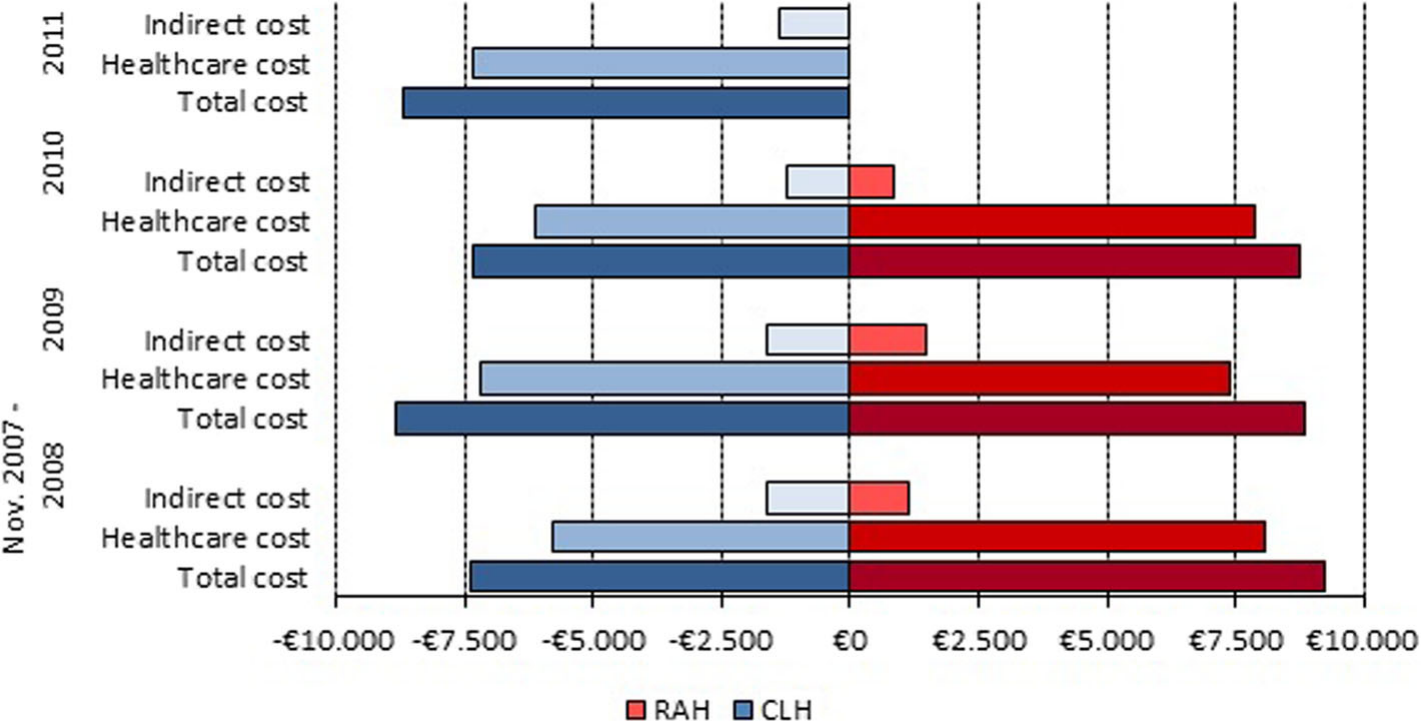
Evolution of cost by year of surgery. Note: In 2011 da Vinci robotic-assisted surgery was not used for surgeries on benign gynecological pathologies

Figure 5 shows the evolution of average healthcare costs by year in which the surgery took place. A downward trend is observed for the RAH group due to lower healthcare costs in 2009 (€703.26; *p* = 0.25) and lower indirect costs in 2010 (€615.51; *p* = 0.003). However, the evolution of the average cost for the CLH group follows no pattern.

Aside from the technique used, the factors associated with increased costs were repeat surgeries and hypertensive patient status. In the RAH group, patients with repeat surgeries had an increase of €7200.61 (*p* < 0.001) in the total cost, €6288.86 of which were healthcare costs and €111.75 indirect costs. In the CLH group, patients with repeat surgeries had a €9553.76 increase in the total cost (*p* < 0.001), €7634.11 of which were healthcare costs and €1919.65 indirect costs.

For hypertensive patients there was a cost increase in both groups. The total cost increase for hypertensive patients in the RAH group was €732.30 (*p* = 0.43), of which €647.10 were healthcare costs and €85.20 indirect costs. The total cost increase for hypertensive patients in the CLH group was €1476.11 (*p* = 0.11), of which €1077.42 were healthcare costs and €398.69 indirect costs.

## Discussion

Since the introduction of the robotic procedures, there has been a push to demonstrate the effectiveness of robotic hysterectomy over the conventional laparoscopic approach. In 2012, Sarlos et al. [2] published a clinical trial comparing surgical outcome and quality of life of robot-assisted laparoscopic hysterectomy with CLH. They concluded that both techniques compare well in most surgical aspects, but the robotic procedure is associated with longer operating times. These findings are comparable with those published in a clinical trial one year later [12]. A systematic review and meta-analysis in women with benign uterine disease determined by randomized studies [2, 5, 12, 13] showed that there are neither statistically significant nor clinically meaningful differences in surgical outcomes between robotic versus laparoscopic hysterectomy. Based on this fact, the authors concluded that robotic surgery was not associated with an improved effectiveness or safety [3].

A clinical guideline initiated by the Danish Health Authority in 2017 [14], conclude that Robot-assisted laparoscopic hysterectomy should only be preferred over conventional laparoscopic hysterectomy after careful consideration because the beneficial effect is uncertain and because of the longer operating time. Nevertheless, a recent updated merged review of two originally separate Cochrane reviews, one on robot-assisted surgery for benign gynecological disease and the other on robot-assisted surgery for gynecological cancer, concluded that evidence on the effectiveness and safety of robot-assisted surgery compared with conventional laparoscopic surgery for benign hysterectomy suggests that surgical complication rates might be comparable [11].

A systematic review conducted by Roh et al. [15], CLH shows significant advantages in total and net operative time, total complication rate and operative cost. Nevertheless this review includes not only gynecologic, but surgery and urology studies. This clinical heterogeneity introduced by integrating various surgical procedures, may contribute to the deviation meta-analyses findings, presumably reflecting the intrinsic properties of each surgical procedure.

It is known that robotic technology is substantially costlier, which is a real concern, considering the increasing pressure to contain costs moving forward. Nevertheless, there is limited evidence of cost analysis studies comparing robotic to laparoscopic hysterectomy for benign indications [16–21] and just 6 studies including abdominal or vaginal approaches [22–27]. Most of those authors conclude that robotic surgical technology appears to be safe and feasible with similar clinical outcomes to open and laparoscopic surgery, and although the clinical evidence of effectiveness is poor, RAH costs are consistently higher than the cost of CLH. In gynecological malignances, some authors have observed a similar total cost when compared robotic, laparoscopy and laparotomic approaches [28].

There are different proposals to reduce the cost, such as increasing the annual caseload to reduce the maintenance cost per case [4, 20, 21]. However, in our experience, while this may lower the cost of acquiring and maintaining the equipment, there is a significant increase in the final cost associated with the reusable material. Increasing surgeon experience is another proposal [20]. In our casuistry, the cost decreases over time, but it is not clear whether the surgeon’s experience is the only contributing factor, since this was not one of the study objectives. Some authors believe that robotic surgery could be more efficient as the surgical procedure becomes more difficult, as i.e. bigger uterine size [21] or complex or highly technical procedures [4]. Even in these cases, the available evidence does not demonstrate that this is actually true, since there are no studies to verify this assertion [29].

Economic evaluation evidence shows that the indirect costs, such as decreased productivity due to disease or death, were excluded from the evaluation [16–18, 20, 21, 30, 31]. In this regard, another innovative contribution of our study is the focus on the societal perspective.

Our findings reveal that robotic surgery reduced the length of hospitalization by 0.90 days and any reduction in the length of hospitalization means the recovery is faster, due that an early incorporation to usual activities after the primary intervention was obtained by patients of RAH group (a difference of 1.62 days). While some authors concur on this point [17, 18, 32, 33], the biggest comparative analysis by Pasic et al. [16] showed no difference in postoperative hospital stay between the robotic hysterectomy group and the CLH group.

We found no studies that considered such relevant healthcare costs as A&E department consultations and specialist consultations. The breakdown of specialist consultations was very similar in both groups, with slightly more visits among the robotic group. This could be because it was an innovative technique it merited closer follow-up on the part of clinicians. On the other hand, there were differences in the breakdown of consultations to the A&E department, where visits by robotic surgery patients were concentrated in the first two years, while visits by conventional laparoscopy patients continued through the fourth year. It could indicate a situation of lower clinical satisfaction on the part of this group of patients.

Although healthcare cost analysis shows no major differences, by introducing the cost of acquiring and maintaining equipment and material, it is evident that the average cost of robotic surgery is €1169 higher per patient.

From a societal perspective, there is a positive aspect of robotic surgery associated with a 2.34-day reduction in productivity loss, the number of trips to the hospital, was higher.

If we consider the total cost, there was an average difference of €967 per patient in favor of CLH. This difference, which is lower than what other authors have found [16–21] is supported by the inclusion of indirect costs.

Unlike other cost-minimization analyses which assume that RAH are equally effective [18], or effectiveness derived from a systematic review of observational studies [31], our study uses this type of economic evaluation after justifying the equivalence between the two techniques, according with evidence of systematic review [11].

A limitation of the study was the not randomized used of the robotic system, because, it is only available once a week and it is shared with other specialties. The use of the robotic system was set by opportunity criteria, using the system when it was available. In addition, although this study design could limit the transferability of the results, our findings are consistent with the evidence of a systematic review [11].

A strength of the study was have the same surgical team in all cases, with similar experience and competence, which reducing the bias in the procedures.

## Conclusion

To our knowledge, this study represents the most expansive analysis of costs associated with robot-assisted laparoscopic hysterectomy for benign disease. Our findings reveal similar effectiveness between RAH and CLH, although CLH is the more efficient option from the point of view of an economic analysis based on cost-minimization.

Furthermore, the use of robotics has a positive impact on both the public health system (reduces the length of surgical procedures and hospital stays) and on the society (lower loss of productivity). Subsequent generations of robots may very well represent the future, but currently it is difficult to justify, from an economic standpoint, the exuberant uptake of robotic surgery for routine hysterectomies. We believe that the lower cost of acquiring and maintaining the equipment should be considered as the primary option for converting this into an efficient procedure, since the impact of other measures has been shown to be insufficient.

## Data Availability

The datasets used and/or analyzed during the current study are available from the first author on reasonable request.

## Abbreviations

A&E: Accident and Emergency
BMI: Body Mass Index
CI: 95% confidence interval
CLH: Conventional laparoscopic hysterectomy
DRG: Diagnosis related groups
RAH: Robot-assisted hysterectomy
RR: Relative risk
